# Infectious Diseases and Clinical Xenotransplantation

**DOI:** 10.3201/eid3007.240273

**Published:** 2024-07

**Authors:** Jay A. Fishman, Nicolas J. Mueller

**Affiliations:** Massachusetts General Hospital and Harvard Medical School, Boston, Massachusetts, USA (J.A. Fishman);; University Hospital Zurich, University of Zurich, Switzerland (N.J. Mueller)

**Keywords:** xenotransplantation, zoonoses, immunocompromised host, immunosuppression, infectious disease, assay development, global warming, viral infection, infection control, emerging infection, United States, Switzerland

## Abstract

Xenotransplantation, transplantation into humans of vascularized organs or viable cells from nonhuman species, is a potential solution to shortages of transplantable human organs. Among challenges to application of clinical xenotransplantation are unknown risks of transmission of animal microbes to immunosuppressed recipients or the community. Experience in allotransplantation and in preclinical models suggests that viral infections are the greatest concern. Worldwide, the distribution of swine pathogens is heterogeneous and cannot be fully controlled by international agricultural regulations. It is possible to screen source animals for potential human pathogens before procuring organs in a manner not possible within the time available for surveillance testing in allotransplantation. Infection control measures require microbiological assays for surveillance of source animals and xenograft recipients and research into zoonotic potential of porcine organisms. Available data suggest that infectious risks of xenotransplantation are manageable and that clinical trials can advance with appropriate protocols for microbiological monitoring of source animals and recipients.

Xenotransplantation, the implantation of vascularized organs or viable cells from nonhuman species into humans, is under development to address the shortage of human organs for transplantation. Clinical xenotransplantation from swine has become more practical through advances in molecular biology (e.g., CRISPR manipulations) that have enabled the breeding of swine with advantageous immunologic traits coupled with newer immunosuppressive regimens (Figure; Appendix Table 1). Recent porcine cardiac and renal transplants survived for about 2 months in hosts with multiple comorbid conditions and who were not candidates for allotransplantation. Decedent xenografts of hearts and kidneys have been used to demonstrate fundamental functions and immune responses of porcine xenografts in human hosts. Prior experience with xenogeneic (pig, bovine) heart valves, tendons, and skin have generally been fixed or sterilized tissues not carrying viable cells. Regulatory guidelines exist for the clinical use of genetically modified animals but incompletely address microbiologic standards (*1*–*5*). Experience in allotransplantation indicates that the risk for xenosis or xenozoonosis (transmission of infection from animals to humans from viable cells of organs or cellular transplantation) is determined by epidemiologic exposure of source animals and human recipients, the net state of immunosuppression, and the underlying factors contributing to infectious risk, including the type, intensity and duration of immunosuppression (*6*,*7*). In human allotransplantation, immunosuppression is largely standardized, the pattern of infections is predictable, and prophylactic regimens are standardized (*6*,*7*). Some infections are considered routine (human cytomegalovirus [CMV], Epstein-Barr virus [EBV]); unexpected infections reflect excess immunosuppression, unusual exposures in the hospital or community, or donor organ–derived infections. Unexpected donor-derived infections are uncommon despite the urgency of screening, given time limitations for organ implantation (*8*,*9*). Data from microbiologic screening studies are often not available until after implantation.

**Figure:**
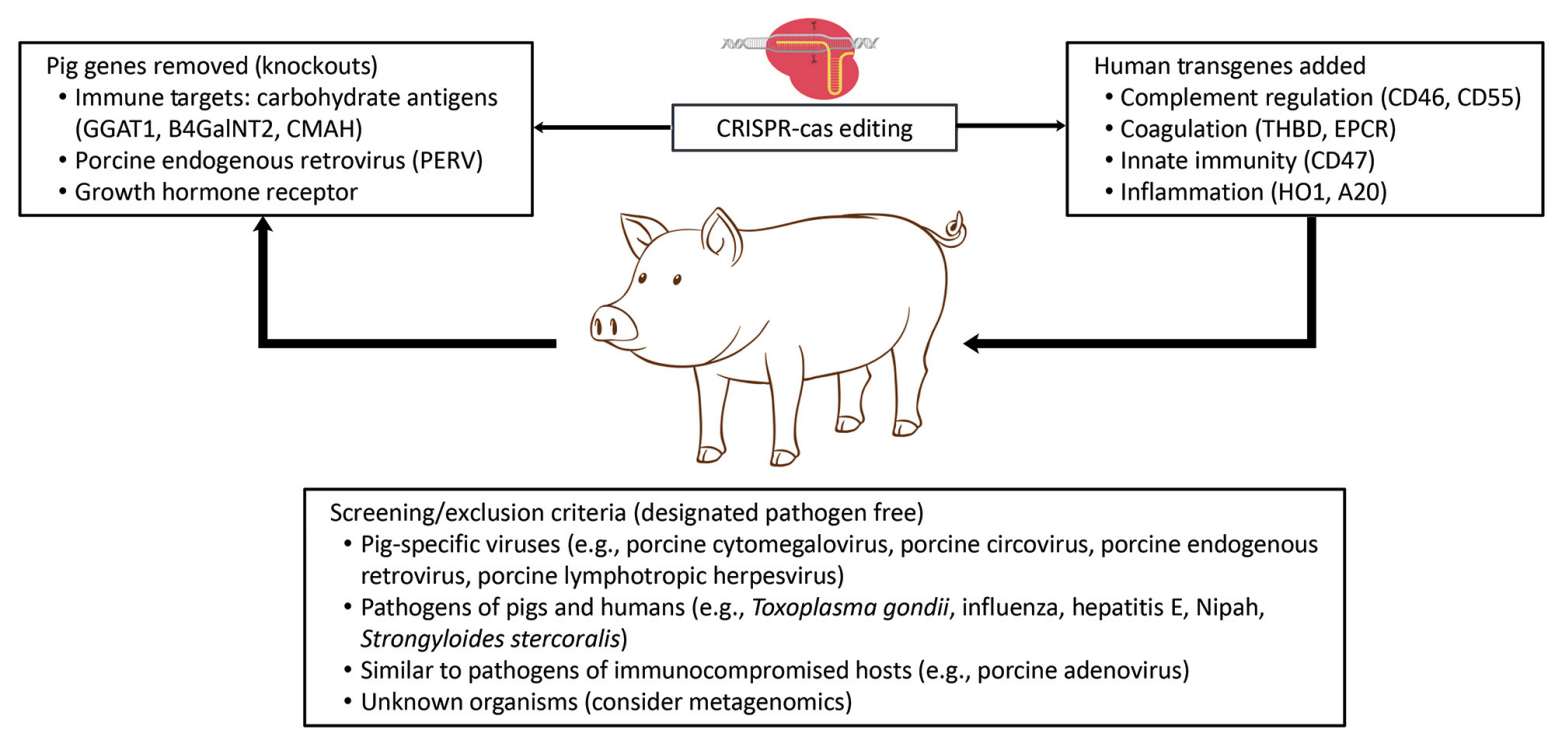
Advances in genetic engineering have led to the breeding of pigs with advantages in infection, immunology, coagulation, size, and inflammation. Breeding of source animals in biosecure facilities enables screening for potential pathogens. B4GalNT2, glycosyltransferase; CD46, human membrane cofactor protein; CD47, block SIRPα tyrosine phosphorylation; CD55, human decay-accelerating factor; CMAH, cytidine monophosphate-N-acetylneuraminic acid hydroxylase; EPCR, endothelial cell protein C receptor; GGAT1, α-1,3-glycosyltransferase; HO1, heme oxygenase-1; HA20, human A20; PERV, porcine endogenous retrovirus; THBD, human thrombomodulin gene.

Immunosuppressed xenograft recipients have potential exposure to microbes carried by xenografts as well as to community-derived exposures and reactivation of latent infections in the human host (*10*). The likelihood of infection caused by any specific organism is unknown, particularly without clinical trials or validated assays for pig-specific pathogens. A challenge and benefit of xenotransplantation is the ability to develop herds of animals free of potential pathogens; thus, developing serologic and molecular assays for use in swine herds and to monitor for infection in recipients is an important component of safety in clinical xenotransplantation.

## Developing Swine as Source Animals for Clinical Xenotransplantation

Consensus does not exist around optimal screening paradigms for source animals and for monitoring of human recipients (Appendix Tables 2, 3). Effective prophylactic strategies require gaining clinical experience, identifying important pathogens, and studying antimicrobial efficacy for organisms from pigs (*10*,*11*). Veterinary guidance for pig health tracks common pathogens and requires screening of animal care providers and animals for infectious exposures. Animals determined specific pathogen–free (SPF) are generally screened for drug-resistant organisms, have limited routine use of antimicrobial agents, are vaccinated extensively, and receive sterile feed in biosecure facilities. Herds of swine for xenotransplantation are maintained in biosecure facilities and monitored routinely to exclude potential human pathogens; this practice is termed designated pathogen-free (DPF) status, a term adopted by regulatory authorities (*12*,*13*). This list of potential pathogens is based on experience in allotransplantation and preclinical xenotransplantation; it includes organisms that cause infection in immunocompromised persons (e.g., *Toxoplasma gondii*) or that are like those causing infection in transplant recipients (Appendix Table 2). Some porcine viruses have known zoonotic potential, including hepatitis E virus, influenza A virus (IAV), Japanese encephalitis virus, and Nipah virus. Pathogens known to be infectious in both pigs and humans (e.g., hepatitis E, influenza) merit monitoring and exclusion from breeding herds (*14*). Depending on the sensitivity of the assays used, organisms excluded from the breeding herd should not pose a threat to xenograft recipients. Pigs are rescreened at the time of organ procurement for xenotransplantation for known pathogens (Appendix Table 3). They are also screened by histopathology, by metagenomic sequencing for unknown organisms, and by routine blood and tissue cultures for bacteria or fungi; not all data are available at the time of procurement.

Immunosuppression for xenotransplantation in nonhuman primates and in recent human xenocardiac and xenokidney recipients has included costimulatory blockade by CD40 or CD154 monoclonal antibodies, mycophenylate mofetil, and T-cell (antithymocyte globulin), B-cell (CD20), and complement inhibition or depletion with perfusion solutions containing anti-inflammatory agents (*15*–*18*). Similar regimens in humans are associated with increased risk for infections from certain viruses (CMV, EBV, BK polyomavirus), *Pneumocystis* spp., *Toxoplasma* spp., and encapsulated bacteria (e.g., *Neisseria meningiditis* A and B).

Groenendaal et al. compiled a list of all known organisms infecting swine from the literature and sorted these based on pathogenicity for pigs and similarity to organisms causing infection in immunocompromised human hosts (*19*). The report identified 254 potential pathogens in pigs in the United States: 108 viruses, 75 bacteria, 59 parasites, 11 fungi and 1 prion; it did not include organisms potentially introduced during the procurement and transportation of organs intended for transplantation (*19*). The list includes organisms important to routine pig health status, pig pathogens, and potential human pathogens; clear distinction is not yet feasible given limited clinical experience (*19*). Most (≈130) pathogens are routinely excluded from biosecure pig-breeding facilities. The list supports development of risk mitigation strategies including requirements for biosecure breeding facilities, pathogen monitoring and exclusion, pharmaceutical treatment or vaccination, and genome editing; however, screening and monitoring for infection remained difficult.

Some viruses identified in preclinical pig-to-primate xenotransplantation appear to be species specific and do not infect human cells; those viruses include porcine cytomegalovirus, PCMV, and porcine circovirus. The viruses proliferate in xenografts in immunosuppressed nonhuman primates (NHP) and may cause graft dysfunction, graft rejection, coagulopathy, or other syndromes (*20*–*23*). In baboon recipients of porcine heart and kidney xenografts, PCMV-infected pig cells and viral DNA are found in circulation despite ganciclovir prophylaxis. PCMV-infected porcine endothelial cells become activated to produce tissue factor, leading to systemic consumptive coagulopathy and accelerated renal xenograft rejection (*12*,*20*,*21*,*23*). PCMV can be excluded from pig colonies by caesarean delivery, early weaning, and biosecure isolation but is easily reintroduced (*24*–*26*) Those interventions inconsistently reduce transmission of porcine lymphotropic herpesvirus (PLHV) from sow to piglet (*25*,*27*). In a human recipient of a porcine cardiac xenograft, PCMV was detected by unbiased plasma microbial cell-free DNA testing despite negative molecular testing of nasal swab and buffy coat before organ procurement and ganciclovir prophylaxis (*28*,*29*). These observations demonstrate the value of pig screening using serologic testing, nucleic acid testing (NAT), and other advanced techniques. Four species of porcine circovirus, 1–4, cause infection in swine; diverse clinical associations have been made between PCVs in swine. PCV2 and PCV3 disseminate with shed cells from cardiac xenografts in baboons; transmission to primate cells has not been demonstrated. No transmission of PCV was identified in a seropositive pig-to-human cardiac recipient (*28*).

The porcine endogenous retroviruses (PERV) have the theoretical capacity to integrate into the hosts’ germline DNA causing insertional effects (*13*,*30*). PERV receptors for PERV-A and -B are ubiquitous in human cells (HuPAR-1 and HuPAR-2) (*31*). PERV-A and PERV-B can infect transformed human cells. PERV-C infects only pig cells. Receptor homologs in porcine cells are active while those in baboon appear inactive; baboons do not have PERV infection develop after xenotransplantation. Recombinant PERV-AC is a naturally occurring recombinant between PERV-A and -C and infects with greater efficiency than PERV-A into transformed human cells via the PERV-A receptor domain. PERV infection of humans exposed to porcine cells has not been reported. Various PERV mitigation strategies for source pigs include selective breeding of PERV-C–free pigs (which does not preclude recipient infection due to PERV-A or PERV-B), or genetic inactivation of the polymerase gene of PERV proviral elements using CRISPR-Cas9 (*32*).

## Shifting Epidemiology of Organisms of Swine

Infectious disease management is a central component of the pork industry. Biosecurity precautions vary across breeding facilities; one precaution is the exclusion of birds, rodents, and amphibians. Strict regulations exist for the international movement of pigs, feed, and pork products. The intensification of animal agriculture, applying technological advances to allow increased density of animal rearing, has accounted for emergence of new zoonoses resulting from various practices including crowded housing, use of antibiotics, deforestation, and inadequate waste management and contributes to global warming (*33*). The spread of animal microbes to humans has increased with contacts between humans and wild or domesticated animal hosts in agriculture and markets (*33*–*35*). For example, the spread of HIV, SARS-CoV-2, Middle East respiratory syndrome virus, swine influenza virus, hepatitis E virus, and Japanese encephalitis virus was the result of contacts between animal reservoirs and humans (*36*–*38*). The need for constant review of pathogens that require surveillance in swine raised for clinical xenotransplantation is demonstrated by porcine circovirus type 4, which was not reported in the literature until 2019 but had been identified in epidemiologic studies of swine for more than a decade (*39*). Global warming and intensified pig farming in previous bat habitats may have contributed to the spread of Nipah virus to swine and farmers in Malaysia. Epidemiologically restricted pathogens of swine are likely to spread to new areas with global warming, economic development, and international travel and trade. These may include many parasites, bacteria such as *Burkholderia* species and viruses such as Nipah, PCV4, lymphocytic choriomeningitis, and Japanese encephalitis. Worldwide, porcine organisms of concern with zoonotic potential are increasing; among those, use of antimicrobial agents is associated with increasing antimicrobial resistance. They include bacteria (*Salmonella*, *S. suis*, *S. aureus*, *Campylobacter*, *Mycobacteria*, *Brucella*, *Leptospira*, *E. coli*), parasites (*Trichinella*, *Toxoplasma*, *Trypanosoma*), and viruses (influenza, Nipah, Japanese encephalitis, Menangel) (*19*). Those pathogens merit surveillance in pig herds as their epidemiologic footprint expands. At the same time, biosecurity facilities have improved through experience and necessity in genetic manipulation and oocyte implantation, which may mitigate some of the challenges of maintenance of DPF status.

## Microbiological Testing in the Human Recipient

The key elements of infection control are exclusion of potential pathogens from breeding herds (DPF status) and monitoring in xenograft recipients and clinical staff (Appendix Table 3) (*11*,*30*,*40*,*41*). Although bacteria, fungi, and parasites can generally be identified in veterinary or clinical microbiologic labs by culture-based techniques, viruses require both serologic and NAT. Multiplexed PCRs against multiple viral targets have been reported for use in pigs (*42*). Pathogen-directed viral assays are not yet validated in humans; some assays may not be able to distinguish between similar porcine and human pathogens (*43*). Porcine retroviruses such as PERV AC have some unstable target sequences or variable tissue tropism and may require functional assays (e.g., reverse transcribed retrovirus on productively infected target cells), full sequence analysis, or in situ hybridization (*44*).

The availability of archived biospecimens from source pigs and recipients, and from persons with significant exposures to donor swine and recipients, will enable researchers to evaluate infections and possible donor-derived transmissions. Metagenomic or next-generation sequencing (NGS) approaches rely on available pathogen sequence data for analyzing sequences derived from animals or in preclinical or clinical recipients. As genetic databases for genomic and microbial sequences grow, retrospective analysis of stored clinical samples is feasible. Because infections are common in immunosuppressed allotransplant recipients, such techniques are also helpful for evaluating infectious syndromes for which a specific diagnosis cannot be established. NGS approaches are pathogen agnostic and may also detect colonizing species or replication-incompetent sequences of unclear clinical significance (*40*). Using a NGS approach is of particular interest for pathogen discovery in the context of xenotransplantation where knowledge of potential porcine pathogens is limited (*45*); the technology was instrumental in the discovery of several new viruses, some associated with human disease (*46*). Those data will also address concerns regarding potential spread of xenogeneic organisms to the general population.

## Prevention and Surveillance of Infection in the Xenograft Recipient

After xenotransplantation, recipient surveillance must consider both swine and human pathogens (Appendix Table 3). Standard allotransplantation prophylactic regimens can be used for perioperative bacterial infections, herpesviruses, molds, *Toxoplasma gondii*, and *Pneumocystis jirovecii*. Novel immunosuppression regimens may alter the spectrum of opportunistic infections. Testing should be guided by knowledge of microbes not excluded from the breeding herd (e.g., PERV and PCMV status). Surveillance will require use of laboratory-developed assays or off-label use of available tests for more extensive pathogen discovery (e.g., NGS). Recent porcine-to-human cardiac and renal xenotransplants successfully used NGS for posttransplant surveillance (*28*). Biopsies used to monitor graft rejection should include microbial analysis using cultures, NGS, immunohistology, and electron microscopy for viral infections. Clinical trials should consider standard protocols for management of fevers or infectious syndromes in addition to routine screening during early periods. Treating graft rejection or infectious syndromes requires increased testing.

## Porcine Antiviral Therapy Prophylaxis and Treatment

Strategies for prevention and treatment of potential viral infections in xenotransplantation, as for allotransplantation, include understanding of the antiviral susceptibilities of porcine viruses. Data on antiviral therapy for porcine viruses are limited (*41*). PCMV does not infect human cells but can provoke graft dysfunction and coagulopathy and will merit prophylaxis and therapy. PCMV has reduced susceptibility to acyclovir, ganciclovir, and foscarnet; ganciclovir prophylaxis at full treatment doses is inconsistently effective in vivo in immunosuppressed NHP xenograft recipients. Consistent with homology with human herpesvirus 6, the nephrotoxic agent cidofovir is more effective at therapeutic concentrations in vitro (*22*,*47*). Porcine lymphotropic herpesviruses (PLHV) 1, 2, and 3 have been associated with a lymphoproliferative disorder after experimental hematopoietic stem cell transplantation in pigs; the viruses are not a known to be pathogens in NHP or in humans, and no effective antiviral drugs exist. PLHV was not activated after xenotransplantation of various organs from swine infected with PLHV into nonhuman primates (*20*)

Regarding PERV, transmission was not identified in decedent recipients of renal xenografts for <72 hours or in recipients of PERV-C negative cardiac xenografts for <60 days; chimerism of cells infected with PERV-A or -B is expected. Retroviral transmission to xenograft recipients remains a concern (*28*,*48*,*49*). Antiretroviral drugs used to treat HIV-1, including reverse transcription inhibitors zidovudine, tenofovir, and adefovir, as well as the integrase inhibitors raltegravir and dolutegravir, can inhibit PERV. Nonnucleoside reverse transcriptase inhibitors (nevirapine) and protease inhibitors lack inhibitory activity for PERV. Should PERV therapy or postexposure prophylaxis be required, combination antiretroviral therapy using integrase inhibitors and active nucleoside reverse transcriptase inhibitors would be recommended.

There are no specific treatments known for circoviruses PCV1–4; however, swine vaccination is available. Caesarean delivery and colostrum deprivation with use of NAT can prevent PCV transmission to piglets.

## Infection Control in Clinical Xenotransplantation

As part of protocol development and the informed consent process, prospective xenograft recipients require education about infectious risks of xenotransplantation to themselves and potentially to social and sexual partners, of which data are limited. In the absence of PERV risk, standard universal precautions for xenograft recipients should be adequate to protect hospital staff and social contacts. No infections have been reported among veterinary staff, scientists, or surgeons participating in preclinical xenotransplant studies. As for any surgical procedure, the risk for exposure is greatest for operating room staff handling pig organs and fluids or via splash or needlestick injury. Standard surgical infection control practices should prevent such exposures. Given the unknowns, archiving baseline leukocyte and plasma samples could enable future investigations should infectious syndromes emerge in xenograft recipients. Additional samples can be obtained for documented exposures to bodily fluids or with undiagnosed infectious syndromes in xenograft recipients or surgical teams. General hospital care workers for xenotransplant recipients should not have risks of exposure beyond those prevented by universal precautions. Infection and infectious syndromes are common in immunosuppressed transplant recipients; recipients should follow isolation precautions based on the primary syndrome (e.g., for diarrhea or pneumonitis).

Occupational health service staff should be aware of xenotransplantation protocols for blood or body fluid exposure from source animals or xenotransplant recipients. In such situations, knowing the infectious status of the source pig and the recipient is invaluable. If the donor animal is PERV negative, postexposure retroviral prophylaxis should not be required. For PERV-positive donors, prophylaxis after needlestick exposure to porcine tissues recommends use of a reverse transcription inhibitor and integrase inhibitor. Testing should include NAT for swine-specific pathogens, as well as standard tests for HIV, hepatitis C and hepatitis B. Repeat NAT testing should be performed at regular intervals (e.g., 1, 3, and 6 months) after a blood or body fluid exposure. Plans for passive surveillance and active testing and treatment will be required for clinical trials; those plans should be developed in conjunction with Infection Control and Occupational Health groups. Informed consent may be required for acquiring and storing blood samples from clinical care providers.

Because clinical experience is limited, infectious risks to close contacts of the xenotransplant recipient are not defined. The clinical trial design and consent process should address the benefits and feasibility of posttransplant surveillance of close contacts to inform blood sample archiving in advance of the procedure in the event of blood or body fluid exposure. As part of pretransplant education, the recipient and close contacts should be instructed to refrain from blood donation and unprotected sexual contacts; household members may be counseled to avoid sharing items that could be contaminated with blood. Education on potential risks to recipients, healthcare providers, and the general public includes ethical considerations for unknown hazards. The actual risk for infectious spread to the public is unknown; most potential pathogens are species specific. Active PERV can be excluded; recombination events should not occur without viral replication but cannot be completely excluded. With careful screening of source animals and monitoring of recipients for unknown as well as known microbes, the risk for xenogeneic spread to the public is very limited. Data from clinical trials will refine our understanding of disease transmission via xenotransplantation and will inform education for potential recipients and the public.

## Conclusions

The risk for transmission of infection due to novel pathogens in association with xenotransplantation is unknown. Microbiological screening of source animals may reduce infectious risk; however, unknown porcine pathogens with capacity to infect humans may exist and are unlikely to be identified in the absence of clinical trials. The effect of the activation of PCMV in 1 cardiac recipient demonstrated the importance of herd screening for xenotransplants (*28*). Studies in deceased recipients of kidneys and hearts have provided information on metabolic and immunologic aspects (e.g., role of innate immunity), but they have reported limited immunosuppression and are of limited durations (<2 months) and so are less informative regarding infectious risks (*50*). Infection control measures include storage of baseline blood samples from the xenograft donor, persons involved in procurement and transplantation of pig organs, and serial monitoring of the recipient and close contacts for known and possible unknown pathogens. Assays, including metagenomics, for potential pig pathogens need to be developed and validated. Transparency is essential in microbiologic investigations performed in clinical xenotransplantation trials.

Appendix Additional information about infectious diseases in xenotransplantation.
