# Supplementary material for: Infectious Diseases and Clinical Xenotransplantation
Source: Emerg Infect Dis. 2024 Jul;30(7):1311–8. doi: 10.3201/eid3007.240273 (PMC11210669; doi:10.3201/eid3007.240273)
Supplement: Appendix — Additional information about infectious diseases in xenotransplantation. [file 24-0273-Techapp-s1.pdf]

Article DOI: http://doi.org/10.3201/eid3007.240273

EID cannot ensure accessibility for supplementary materials supplied by authors. Readers who have difficulty accessing supplementary content should contact the authors for assistance.

Infectious Diseases and Clinical Xenotransplantation

Appendix

Appendix Table 1. Potential targets for genetic manipulation in swine xenograft donors

Target type	Target	Potential gene targets for xenotransplantation
Pig breed	—	Various pig breeds
Endogenous retrovirus Inactivation	Virus	Porcine endogenous retrovirus (PERV A, B, C, AC)
Knockout	Carbohydrate antigens	GGAT1 (α-1,3-glycosyltransferase)
	Carbohydrate antigens	B4GalNT2 (glycosyltransferase)
	Carbohydrate antigens	CMAH (cytidine monophosphate-N-acetylneuraminic acid hydroxylase)
	Organ growth	Growth hormone receptor
Added human transgenes	Complement regulation	CD46 (hMCP, human membrane cofactor protein)
	Complement regulation	CD55 (hDAF human decay-accelerating factor)
	Coagulation	THBD (human thrombomodulin gene)
	Coagulation	EPCR (Endothelial cell protein C receptor)
	Innate immunity	CD47 (Block SIRPα tyrosine phosphorylation)
	Inflammation, apoptosis	HO1 (Heme Oxygenase-1)
	Inflammation, apoptosis	HA20 (Human A20)

Appendix Table 2. Risk categories for potential pathogens in recipients of porcine xenografts (*1*,*2*)*

Organism hosts	Examples	Microbiologic assays available?	Monitor in breeding colony?
Pathogens of immunologically normal humans and swine	Influenza viruses* (*3–6*); Hepatitis E virus* (*7–9*); *Mycobacterium tuberculosis**; rabies*; many bacterial and parasitic species (e.g., Ascaris, Toxocara, Pasteurella multocida; Mycoplasma spp.)	Yes	Yes
Known pathogens of immunosuppressed human transplant recipients	*Toxoplasma gondii*; Strongyloides* spp.*; *Aspergillus sp.; Cryptococcus spp; Cryptosporidium spp.*	Yes	Based on risk with organism
Porcine organisms similar to common pathogens of immunosuppressed human transplant recipients	Porcine adenovirus; porcine parvovirus 1; porcine respiratory coronavirus; parainfluenza virus 3	Few	Require validation of assays in human blood or tissues.
Unique swine pathogens (may replicate only in pig cells)	Porcine cytomegalovirus (PCMV)* (*14–18*); Porcine circovirus (PCV 1–4) (*19–24*); porcine lymphotropic herpesvirus (PLHV 1,2); porcine endogenous retrovirus* (PERV A, B, C, AC) (*1*,*25*)	Some	Herpesviruses generally species-specific. Risk requires clinical study
Organisms routinely tested for health status of swine	Porcine enterovirus spp; *Lawsonia Intracellularis;* porcine epidemic diarrhea virus;transmissible gastroenteritis virus; porcine delta coronavirus; *Brucella suis*; porcine reproductive and respiratory syndrome virus; porcine epidemic diarrhea virus;*Pseudorabies* virus	Yes	Yes

Organism hosts	Examples	Microbiologic assays available?	Monitor in breeding colony?
Porcine organisms largely geographically restricted (*4*,*26*,*27*) (examples)	*Burkholderia pseudomallei*; *Clonorchis sinensis; Echinococcus* spp; *Schistosoma* spp; African swine fever (ASF) virus; Menangle virus; Nipah virus (*28*,*29*); porcine circovirus type 4 (PCV4)	Some	Monitor for future geographic spread

*Consider exclusion of infected animals carrying these species. PERV may be excluded genetically (e.g., CRISPR-cas9) ; PERV-C negative animals carry potentially infectious PERV-A and B.

Appendix Table 3. Considerations in routine testing of xenograft recipients*

Virus	Testing method
Porcine endogenous retrovirus (PERV) A, B, C, AC (if present in source animal) +	Qualitative and quantitative (QNAT) nucleic acid testing (NAT); antibody- based tests (serology, ELISA, Western Bylot)+
Porcine lymphotropic herpesvirus type 2 (PLHV-1–2)	QNAT+
Porcine circovirus (PCV 1–4)	QNAT
Porcine cytomegalovirus (PCMV)	NAT+; serology
Human cytomegalovirus (HCMV) – per risk status	QNAT, serology
Human Epstein-Barr virus (EBV) – per risk status	QNAT, serology
BK polyomavirus (kidney recipients) – per protocol	QNAT
Pig cell chimerism in circulation (PBMC)	QNAT+ (e.g., P-MHC class I gene; p-mtCOII gene) in recipient PBMC DNA.
Unknown pathogens	Metagenomics or next generation sequencing (*10*–*13*)

* Additional testing is needed for individuals with infectious syndromes. QNAT: quantitative nucleic acid test; P-MHC: porcine major histocompatibility complex; p-mtCOII: pig mitochondrial cytochrome *c* oxidase subunit II gene
+ Quantitative NAT for PERV and other viruses must be normalized against chimerism studies to correct for the number of circulating pig cells in blood samples.

References

1. Fishman JA. Infectious disease risks in xenotransplantation. Am J Transplant. 2018;18:1857–64. PubMed https://doi.org/10.1111/ajt.14725

2. Fishman JA. Risks of Infectious Disease in Xenotransplantation. N Engl J Med. 2022;387:2258–67. PubMed https://doi.org/10.1056/NEJMra2207462

3. Campitelli L, Donatelli I, Foni E, Castrucci MR, Fabiani C, Kawaoka Y, et al. Continued evolution of H1N1 and H3N2 influenza viruses in pigs in Italy. Virology. 1997;232:310–8. PubMed https://doi.org/10.1006/viro.1997.8514

4. Lopez-Moreno G, Davies P, Yang M, Culhane MR, Corzo CA, Li C, et al. Evidence of influenza A infection and risk of transmission between pigs and farmworkers. Zoonoses Public Health. 2022;69:560–71. PubMed https://doi.org/10.1111/zph.12948

5. Webster RG, Bean WJ, Gorman OT, Chambers TM, Kawaoka Y. Evolution and ecology of influenza A viruses. Microbiol Rev. 1992;56:152–79. PubMed https://doi.org/10.1128/mr.56.1.152-179.1992

6. Zhou NN, Senne DA, Landgraf JS, Swenson SL, Erickson G, Rossow K, et al. Genetic reassortment of avian, swine, and human influenza A viruses in American pigs. J Virol. 1999;73:8851–6. PubMed https://doi.org/10.1128/JVI.73.10.8851-8856.1999

7. Fang SY, Han H. Hepatitis E viral infection in solid organ transplant patients. Curr Opin Organ Transplant. 2017;22:351–5. PubMed https://doi.org/10.1097/MOT.0000000000000432

8. Meng XJ. Swine hepatitis E virus: cross-species infection and risk in xenotransplantation. Curr Top Microbiol Immunol. 2003;278:185–216. PubMed https://doi.org/10.1007/978-3-642-55541-1_7

9. Wang B, Meng XJ. Hepatitis E virus: host tropism and zoonotic infection. Curr Opin Microbiol. 2021;59:8–15. PubMed https://doi.org/10.1016/j.mib.2020.07.004

10. Casto AM, Fredricks DN, Hill JA. Diagnosis of infectious diseases in immunocompromised hosts using metagenomic next generation sequencing-based diagnostics. Blood Rev. 2022;53:100906. https://doi.org/10.1016/j.blre.2021.100906

11. Greninger AL. The challenge of diagnostic metagenomics. Expert Rev Mol Diagn. 2018;18:605–15. PubMed https://doi.org/10.1080/14737159.2018.1487292

12. Santiago-Rodriguez TM, Hollister EB. Unraveling the viral dark matter through viral metagenomics. Front Immunol. 2022;13:1005107. PubMed https://doi.org/10.3389/fimmu.2022.1005107

13. Simner PJ, Miller S, Carroll KC. Understanding the promises and hurdles of metagenomic next-generation sequencing as a diagnostic tool for infectious diseases. Clin Infect Dis. 2018;66:778–88. PubMed https://doi.org/10.1093/cid/cix881

14. Mueller NJ, Barth RN, Yamamoto S, et al. Activation of cytomegalovirus in pig-to-primate organ xenotransplantation. J Virol. 2002;76:4734–40.

15. Gollackner B, Mueller NJ, Houser S, Qawi I, Soizic D, Knosalla C, et al. Porcine cytomegalovirus and coagulopathy in pig-to-primate xenotransplantation. Transplantation. 2003;75:1841–7. PubMed https://doi.org/10.1097/01.TP.0000065806.90840.C1

16. Yamada K, Tasaki M, Sekijima M, Wilkinson RA, Villani V, Moran SG, et al. Porcine cytomegalovirus infection is associated with early rejection of kidney grafts in a pig to baboon xenotransplantation model. Transplantation. 2014;98:411–8. PubMed https://doi.org/10.1097/TP.0000000000000232

17. Sekijima M, Waki S, Sahara H, Tasaki M, Wilkinson RA, Villani V, et al. Results of life-supporting galactosyltransferase knockout kidneys in cynomolgus monkeys using two different sources of galactosyltransferase knockout Swine. Transplantation. 2014;98:419–26. PubMed https://doi.org/10.1097/TP.0000000000000314

18. Denner J, Längin M, Reichart B, Krüger L, Fiebig U, Mokelke M, et al. Impact of porcine cytomegalovirus on long-term orthotopic cardiac xenotransplant survival. Sci Rep. 2020;10:17531. PubMed https://doi.org/10.1038/s41598-020-73150-9

19. Allan GM, McNeilly F, Kennedy S, et al. Isolation of porcine circovirus-like viruses from pigs with a wasting disease in the USA and Europe. J Vet Diagn Invest. 1998;10:3–10. https://doi.org/10.1177/104063879801000102

20. Denner J, Mankertz A. Porcine circoviruses and xenotransplantation. Viruses. 2017;9:83. PubMed https://doi.org/10.3390/v9040083

21. Klaumann F, Correa-Fiz F, Franzo G, Sibila M, Núñez JI, Segalés J. Current knowledge on *Porcine circovirus 3* (PCV-3): a novel virus with a yet unknown impact on the swine industry. Front Vet Sci. 2018;5:315. PubMed https://doi.org/10.3389/fvets.2018.00315

22. Krüger L, Längin M, Reichart B, Fiebig U, Kristiansen Y, Prinz C, et al. Transmission of porcine circovirus 3 (PCV3) by xenotransplantation of pig hearts into baboons. Viruses. 2019;11:650. PubMed https://doi.org/10.3390/v11070650

23. Zhai SL, Lu SS, Wei WK, Lv DH, Wen XH, Zhai Q, et al. Reservoirs of porcine circoviruses: a mini review. Front Vet Sci. 2019;6:319. PubMed https://doi.org/10.3389/fvets.2019.00319

24. Zhang H-H, Hu W-Q, Li J-Y, Liu TN, Zhou JY, Opriessnig T, et al. Novel circovirus species identified in farmed pigs designated as Porcine circovirus 4, Hunan province, China. Transbound Emerg Dis. 2020;67:1057–61. PubMed https://doi.org/10.1111/tbed.13446

25. Fishman JA, Patience C. Xenotransplantation: infectious risk revisited. Am J Transplant. 2004;4:1383–90. PubMed https://doi.org/10.1111/j.1600-6143.2004.00542.x

26. McLean RK, Graham SP. The pig as an amplifying host for new and emerging zoonotic viruses. One Health. 2022;14:100384. PubMed https://doi.org/10.1016/j.onehlt.2022.100384

27. Pavio N, Meng XJ, Renou C. Zoonotic hepatitis E: animal reservoirs and emerging risks. Vet Res. 2010;41:46. PubMed https://doi.org/10.1051/vetres/2010018

28. Drew TW. The emergence and evolution of swine viral diseases: to what extent have husbandry systems and global trade contributed to their distribution and diversity? Rev Sci Tech. 2011;30:95–106. PubMed https://doi.org/10.20506/rst.30.1.2020

29. VanderWaal K, Deen J. Global trends in infectious diseases of swine. Proc Natl Acad Sci U S A. 2018;115:11495–500. PubMed https://doi.org/10.1073/pnas.1806068115
